# Oxr1 and Ncoa7 regulate V-ATPase to achieve optimal pH for glycosylation within the Golgi apparatus and trans-Golgi network

**DOI:** 10.1073/pnas.2505975122

**Published:** 2025-05-30

**Authors:** Shin-ichiro Yoshimura, Tomoaki Sobajima, Masataka Kunii, Akihiro Harada

**Affiliations:** ^a^Department of Cell Biology, Graduate School of Medicine, The University of Osaka, Suita 565-0871, Osaka, Japan

**Keywords:** Rab, V-ATPase, Golgi apparatus/Trans-Golgi network, glycosylation, congenital disorders of glycosylation

## Abstract

Maintenance of pH within membranous organelles is crucial for cellular processes such as posttranslational modifications, ligand–receptor interactions, and proteostasis. The precise mechanisms that determine the luminal pH of each organelle are not fully understood. This study investigated the mechanisms that regulate luminal pH to ensure optimal enzymatic activity. We identified Oxr1 and its paralog Ncoa7, which regulate the vacuolar-type proton pump ATPase (V-ATPase) at the Golgi apparatus and trans-Golgi network (TGN). Oxr1 and Ncoa7 were predominantly localized at the Golgi and TGN membranes, dependent on their binding to various GTP-bound Rab proteins. In vitro experiments using purified recombinant proteins indicated that Oxr1 and Ncoa7 directly bind to the catalytic subunit of V-ATPase, inhibiting its ATP hydrolytic activity via their TLDc domains. We observed significant acidification of the Golgi/TGN lumen in Oxr1- and Ncoa7-depleted cells. Lectin blot analysis demonstrated that depletion of Oxr1 and Ncoa7 led to a defect in protein glycosylation, a major enzymatic posttranslational modification in the Golgi and TGN. Furthermore, depletion of Oxr1 and Ncoa7, along with drug-induced inhibition of glycosylation, increased lysosomal pH and sensitivity to silicon dioxide-induced membrane damage. This apparent lysosomal dysfunction suggested that, in addition to the Golgi and TGN, Oxr1 and Ncoa7 also contribute to the integrity of other organelles. Our findings indicate that Oxr1 and Ncoa7 protect the Golgi and TGN lumen from excess acidification by inhibiting V-ATPase activity and providing an optimal environment for enzymatic activity in the Golgi and TGN.

## Significance

In the secretory pathway, the pH within organelles is gradually acidified, starting at 7.1 to 7.2 in the endoplasmic reticulum, decreasing to 6.0 to 6.7 in the Golgi apparatus/trans-Golgi network (Golgi/TGN), and reaching 4.5 to 5.0 in lysosomes. Acidic pH is maintained by the vacuolar-type ATPase (V-ATPase) present at the Golgi, lysosomes, and other organelles in the late secretory and endocytic pathways; however, the mechanism underlying this pH variation within different organelles has not been elucidated. Here, we identified the TLDc domain-containing proteins Oxr1 and Ncoa7 as Rab-binding proteins that localize to the Golgi/TGN membrane and regulate luminal pH by inhibiting V-ATPase activity. Our findings explain why the pH in the Golgi/TGN is less acidic than that in lysosomes.

In the secretory pathway, newly synthesized soluble and transmembrane proteins are initially translocated to the endoplasmic reticulum (ER). Folded and core glycosylated proteins in the ER are subsequently delivered to the Golgi apparatus and processed by Golgi-resident glycosidases and glycosyltransferases. These proteins are then sorted through the trans-Golgi network (TGN) and delivered to their final destinations, which include endosomes, lysosomes, and the plasma membrane. This protein transport process participates in compartmentalization and control of the luminal environment of each organelle, defining its identity (1).

The small guanine nucleotide-binding Rab proteins regulate various steps of membrane traffic events through their specific effector proteins. Each of the 60-plus Rab proteins encoded in the mammalian genome is localized to a specific organelle, where its role in regulating vesicle transport may define the identity of the organelle (1, 2). However, the precise roles of the numerous Rab and effector proteins have not yet been fully explored.

The variation in luminal pH among organelles is a key determinant of their function, directly influencing the maximum activities of glycosidases and glycosyltransferases in the Golgi and TGN (pH: 6.0 to 6.7) and hydrolases in lysosomes (pH 4.5 to 5.0) (3). Although the acidic environment is thought to be primarily controlled by several ion transporters, channels, and proton pump ATPases, the precise mechanism that determines the luminal pH of each acidic organelle is not fully understood.

The Tre2/Bub2/Cdc16 (TBC), lysine motif (LysM), and domain catalytic (TLDc) family is highly conserved in eukaryotes, comprising five members in mammalian cells: Ncoa7, Oxr1, TBC1D24, TLDC1, and TLDC2 (4). Accumulating evidence has shown that some TLDc proteins are associated with the cytosolic V_1_ sector of vacuolar-type proton pump ATPase (V-ATPase) and function as regulators of V-ATPase (5–7). However, the organellar functions of these five TLDc proteins and their regulation of V-ATPase activity remain unclear. Here, we show that two TLDc-containing paralogs, Oxr1 and Ncoa7, localize to the Golgi and TGN in a Rab-dependent manner and negatively regulate V-ATPase activity, thereby creating an optimal environment for the activities of Golgi- and TGN-resident glycoenzymes. Our findings shed new light on the roles of Rab and TLDc proteins in organellar pH homeostasis.

## Results

### Identification of Oxr1 and Ncoa7 as Rab-Binding Proteins.

**Fig. 1.**
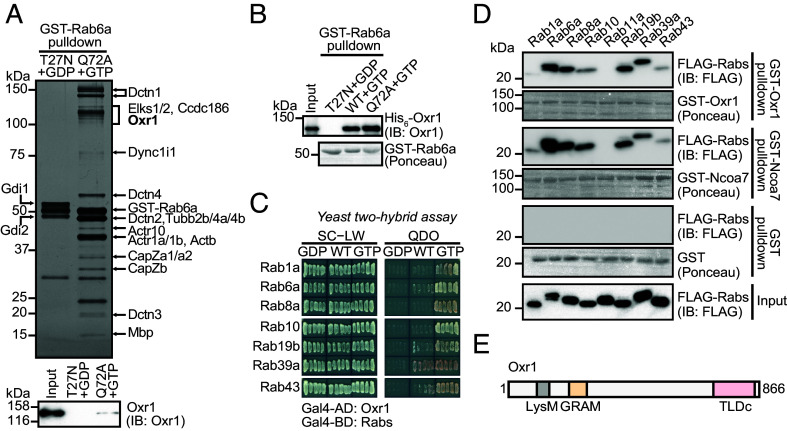
Oxr1 and Ncoa7 binds Rab proteins. (*A*) Silver-stained SDS-PAGE of pulled down proteins with immobilized GST-fused GDP-bound Rab6a T27N or GTP-bound Rab6a Q72A proteins (*Top*). The pulled down proteins were analyzed by mass spectrometry. Immunoblotting with anti-Oxr1 antibody confirming that the Q72A+GTP sample contained Oxr1 (*Bottom*). (*B*) Immunoblotting analysis of pulled down purified His_6_-tagged Oxr1 protein with immobilized GST-fused GDP-bound Rab6a T27N, GTP-bound Rab6a WT, or Q72A proteins. The pulled down Oxr1 was analyzed using anti-Oxr1 antibody (*Top*). The pulled down Rab6a proteins were indicated by Ponceau S staining (*Bottom*). (*C*) Yeast two-hybrid assay using Oxr1 and WT, GTP-, or GDP-restricted mutants of Rab in yeast grown on SC-LW or QDO media. See also *SI Appendix*, Fig. S1. (*D*) SDS-PAGE and immunoblotting analysis of pulled down FLAG-fused Rab proteins from HEK293 cell lysate with immobilized GST, GST-Oxr1, or GST-Ncoa7 proteins. The pulled down Rab proteins were analyzed using an anti-FLAG antibody, and the bound GST, GST-Oxr1, and GST-Ncoa7 proteins were indicated by Ponceau S staining. FLAG-Rab11a was used as a negative control. (*E*) Diagram of the domain structure of Oxr1, indicating the LysM, GRAM, and TLDc domains.

Our initial aim was to identify previously unknown Rab6-binding proteins. Using a glutathione S-transferase (GST) pull-down assay with mouse brain lysates, we identified Oxr1 as a GTP-bound Rab6-binding protein, in addition to known Rab6-binding proteins (e.g., Elks1/2) and subunits of the dynein–dynactin complex (Dync1i1, Dctn1, Dctn2, Dctn3, Dctn4, Actb, Actr1, Actr10, CapZa, and CapZb) (Fig. 1*A*). Additionally, we detected a known Rab2-binding protein, Ccdc186 (Fig. 1*A*) (8–12), implying that it may bind to multiple Rab proteins (Fig. 1*A*). In a pull-down experiment using recombinant proteins, Oxr1 showed direct binding to GTP-loaded wild-type (WT) Rab6 and a GTP-restricted Q72A mutant, but not the GDP-loaded T27N mutant of Rab6 (Fig. 1*B*). Because Oxr1 and its paralog Ncoa7 were previously reported to bind Rab8 and Rab10 (9), we further examined the binding specificities of 58 GTP-restricted Rab mutants using a yeast two-hybrid assay. The results revealed that Oxr1 bound Rab1, Rab19b, Rab39a, and Rab43, in addition to Rab6, Rab8, and Rab10 (*SI Appendix*, Fig. S1*A*), showing slight binding to the WT proteins and strong binding to GTP-restricted, but not GDP-restricted, mutants of these Rab proteins (Fig. 1*C* and *SI Appendix*, Fig. S1*B*). These interactions were confirmed by GST pull-down assays using recombinant GST-fused Oxr1 and Ncoa7 proteins with cell lysates expressing FLAG-tagged GTP-restricted Rab mutants (Fig. 1*D*). These data suggested that Oxr1 and Ncoa7 bind to various GTP-bound Rab proteins.

Oxr1 and Ncoa7 possess a LysM and a GRAM domain at the N-terminus, and a TLDc domain at the C-terminus (Fig. 1*E*) (13). GRAM domains bind to phosphoinositides (PIPs) on organelle membranes (14), while TLDc domains mediate associations with V-ATPases (6, 15, 16). To determine the region necessary for binding to Rab proteins, we tested a series of Oxr1 deletion constructs using a yeast two-hybrid assay. Our data indicated that all Rab proteins generally bind to a region of Oxr1 between the LysM and the TLDc domain, with only Rab19b possessing two separate binding regions (*SI Appendix*, Fig. S1 *C* and *D*).

### Oxr1 and Ncoa7 Localize at the Golgi Apparatus and TGN.

**Fig. 2.**
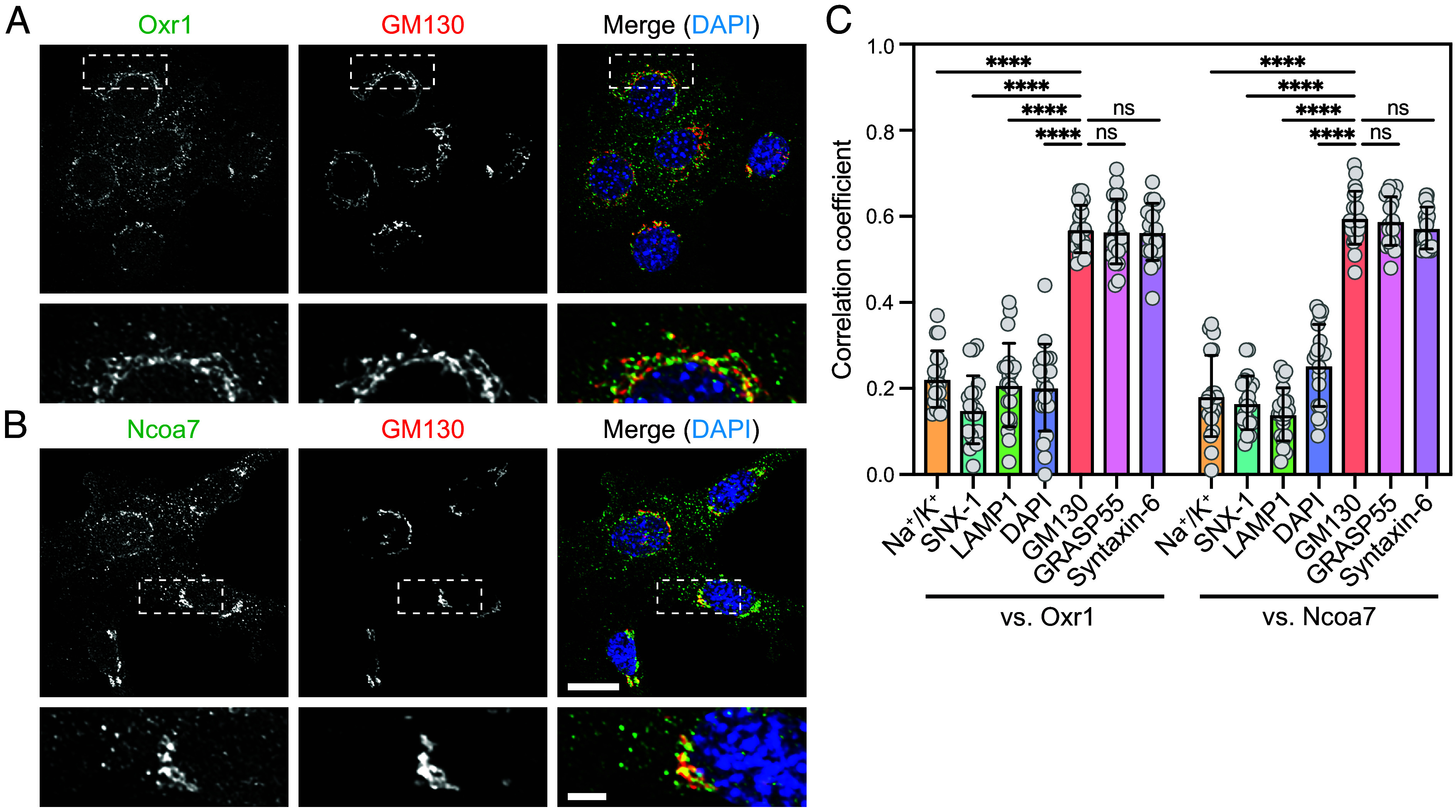
Subcellular localization of Oxr1 and Ncoa7. (*A* and *B*) Costaining of EpH4 cells with anti-GM130 and anti-Oxr1 (*A*) or anti-Ncoa7 (*B*) antibodies, with bottom panels showing magnification of the areas indicated by dashed lines. [Scale bars, 20 µm (main image); 5 µm (magnified image).] (*C*) Quantification of colocalization analyses of Oxr1 or Ncoa7 with GM130 (cis-Golgi) GRASP55 (medial-Golgi), Syntaxin-6 (TGN), LAMP1 (LE/Lys), DAPI (nucleus), SNX1 (sorting endosomes), and Na^+^/K^+^ ATPase (plasma membrane); n = 20 individual cells from three independent fields per experiment. Data in graphs expressed as means ± SD. Statistical significance analyzed using the two-tailed Student’s *t* test (****P < 0.001; ns, not significant). See also *SI Appendix*, Figs. S2 and S3.

We examined the localization of endogenous Oxr1 and Ncoa7 in the mouse mammary epithelial cell line EpH4 using antibody costaining. Oxr1 and Ncoa7 showed significant, strong overlap with the Golgi apparatus, marked by GM130 and GRASP55, and the TGN, indicated by Syntaxin-6 (Fig. 2*C* and *SI Appendix*, Figs. S2 and S3*A*). There was weaker overlap with LAMP1-labeled late endosomes/lysosomes (LE/Lys), Na^+^/K^+^ ATPase-labeled plasma membrane, and sorting nexin-1 (SNX1)-labeled endosomes (Fig. 2*C* and *SI Appendix*, Fig. S3 *C* and *D*). Only a small population of Oxr1 and Ncoa7 molecules were colocalized with SNX1-positive endosomes (Fig. 2*C* and *SI Appendix*, Fig. S3*B*, arrowheads). The signal obtained by co-staining for Oxr1 and Ncoa7 was abolished in Oxr1/Ncoa7-depleted (Oxr1^KO^/Ncoa7^KD^) cells (*SI Appendix*, Fig. S2; see Fig. 5*A* for depletion efficiency), confirming that the antibodies specifically detect endogenous Oxr1 and Ncoa7. We also observed the Golgi localization of exogenously expressed Oxr1 fused with enhanced green fluorescent protein (EGFP) (*SI Appendix*, Fig. S4). Using a mouse neuroblastoma cell line, Neuro-2A, immunofluorescence analysis showed Ncoa7 in the perinuclear region, overlapping with GRASP55 (*SI Appendix*, Fig. S5*A*), but no detectable expression of Oxr1 (*SI Appendix*, Fig. S5*B*). The Golgi colocalization of Oxr1 and Ncoa7 was further confirmed by subcellular fractionation using density gradient ultracentrifugation. Oxr1 and Ncoa7 were mostly detected in the cytosolic fraction, but significant amounts of these proteins cofractionated with the Golgi (GM130) and recycling endosomes (transferrin receptor, TfnR), showing clear separation from the LE/Lys (LAMP1), ER (Calnexin), and mitochondria (COXIV) fractions (*SI Appendix*, Fig. S6).

**Fig. 3.**
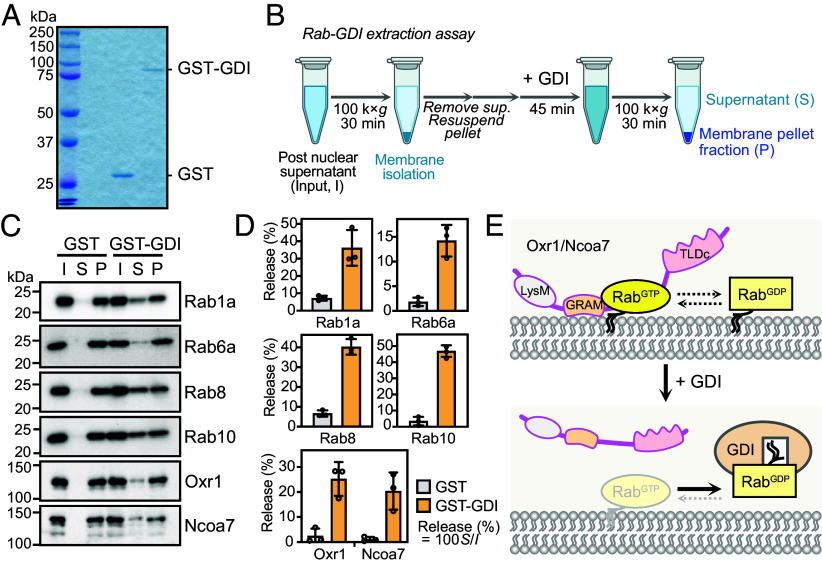
Membrane binding of Oxr1 and Ncoa7 is Rab-dependent. (*A*) Coomassie brilliant blue-stained gel of the recombinant proteins used for the Rab-GDI extraction assay. (*B*) Illustration outlining the Rab-GDI extraction assay. (*C*) Immunoblotting analysis of the membrane fraction of EpH4 cells treated with recombinant GST or GST-Rab GDP dissociation inhibitor (GST-GDI) in GDP-containing buffer to release Rab protein from the membrane (input, I), as well as samples separated into supernatant (S) and precipitated membrane fraction (P), with detection of the membrane-released endogenous Rab, Oxr1, and Ncoa7 proteins using the indicated antibodies. (*D*) Percentages of released Rab, Oxr1, and Ncoa7 proteins per input. Data expressed as means ± SD (n = 3 independent experiments). (*E*) Illustration depicting the Rab-GDI-dependent extraction of Rab proteins triggering the release of Oxr1/Ncoa7 from the membrane.

Next, we investigated whether the binding of Oxr1/Ncoa7 to membranes is Rab-dependent. Isolated membranes were incubated with recombinant GST or the GST-fused Rab GDP dissociation inhibitor (GST-GDI) protein in the presence of GDP (Fig. 3 *A* and *B*), then separated into supernatant and membrane pellets by ultracentrifugation (Fig. 3*B*). Rab proteins were extracted from the membrane in the presence of GST-GDI, and membrane-bound Oxr1 and Ncoa7 were concomitantly released from the membrane (Fig. 3 *C*–*E*). Taken together, these results indicated that both Oxr1 and Ncoa7 localize to the Golgi/TGN in a Rab-dependent manner.

### Oxr1 and Ncoa7 Negatively Regulate V-ATPase Activity Through Direct Interaction with ATP6V1A.

**Fig. 4.**
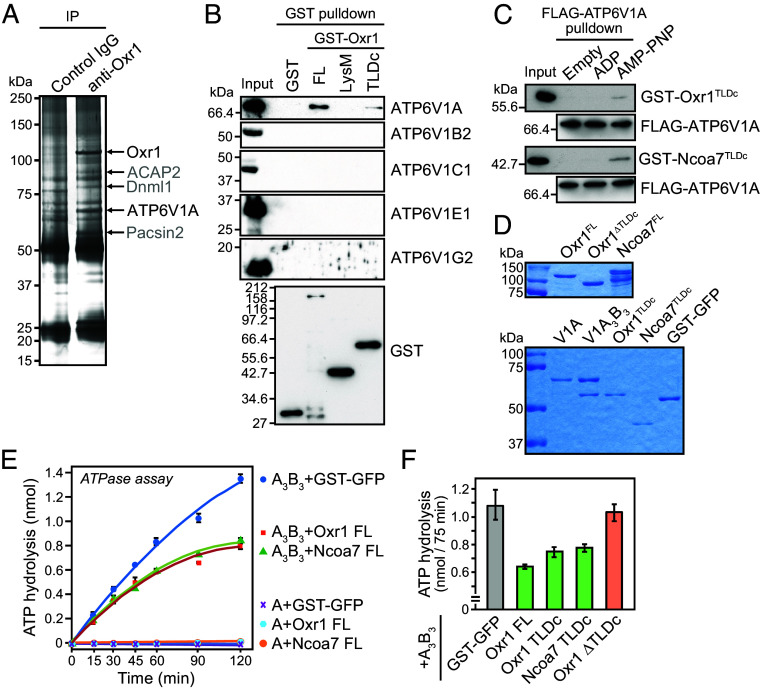
Oxr1 and Ncoa7 inhibit V-ATPase activity. (*A*) Silver-stained SDS-PAGE of immunoprecipitated proteins with control IgG or anti-Oxr1 antibodies. Coprecipitated protein bands were analyzed by mass spectrometry. See also *SI Appendix*, Table S1. The identified proteins are indicated by arrows. (*B*) SDS-PAGE and immunoblotting analysis of pulled down proteins with immobilized recombinant GST, GST-Oxr1 full-length (FL), GST-Oxr1-LysM, and GST-Oxr1-TLDc proteins, using antibodies against V-ATPase V_1_ subunits (ATP6V1A, B2, C1, E1, and G2) and GST. See also *SI Appendix*, Table S2 for the mass spectrometric data of the pull-down experiment with full-length GST-Oxr1. (*C*) Pull-down assay of the immobilized FLAG-tagged ATP6V1A subunit purified from HEK293 cells and loaded with ADP or AMP-PNP, then incubated with the GST-Oxr1^TLDc^ or GST-Ncoa7^TLDc^ mutants. The interaction of Oxr1^TLDc^ or Ncoa7 Ncoa7^TLDc^ with FLAG-ATP6V1A was detected by immunoblotting using anti-GST antibody. (*D*) Coomassie brilliant blue-stained gel of the recombinant proteins used for the ATPase assay. (*E*) ATP hydrolysis of A subunit or A_3_B_3_ complex of *T. thermophilus* V-ATPase in reactions containing His_6_-tagged GST-GFP, Oxr1, or Ncoa7 proteins. (*F*) Effects of GST-GFP, His_6_-Oxr1, GST-Oxr1 TLDc, GST-Ncoa7 TLDc, and His_6_-Oxr1 (∆TLDc) on the ATP hydrolysis activity of V-ATPase.

To further explore the functions of Oxr1 and Ncoa7, we performed coimmunoprecipitation of mouse brain extracts using an anti-Oxr1 antibody. Several proteins coprecipitated with Oxr1, including the A subunit of the V_1_ sector of V-ATPase known as ATP6V1A (Fig. 4*A* and *SI Appendix*, Table S1), which has been reported to associate with Oxr1 and Ncoa7 (17). Our immunoprecipitation and mass spectrometry analyses identified only ATP6V1A among the eight V_1_ sector subunits, indicating that the Oxr1–ATP6V1A interaction has the highest affinity (*SI Appendix*, Table S1). To confirm this, we performed a GST pull-down assay using GST-fused full-length Oxr1 or Oxr1-deletion constructs with brain lysates prepared in RIPA buffer to dissociate the subunits from the V-ATPase complex. The data showed that GST-Oxr1 interacted with ATP6V1A through the TLDc domain, but we were unable to detect the interaction with other subunits (Fig. 4*B* and *SI Appendix*, Table S2). We further tested the direct interaction between the Oxr1- or Ncoa7-derived TLDc domain and ATP6V1A using recombinant proteins. Notably, GST-TLDc domain proteins specifically bound to ATP6V1A loaded with AMP-PNP, a nonhydrolyzable ATP analog, but not to nucleotide-free or ADP-loaded ATP6V1A (Fig. 4*C*). These findings led us to test whether Oxr1 and Ncoa7 directly regulate V-ATPase activity. Because the active catalytic subunit of mammalian V-ATPase was difficult to purify, we performed an in vitro ATPase assay using purified recombinant active A_3_B_3_ hexamer or the sole A subunit of *Thermus thermophilus* V-ATPase (Fig. 4*D*). This assay demonstrated ATPase activity for the A_3_B_3_ hexamer, but not for the A subunit alone (Fig. 4*E*; compare A_3_B_3_+GST-GFP with A + GST-GFP). Furthermore, the ATPase activity of A_3_B_3_ was inhibited in the presence of purified full-length Oxr1 or Ncoa7, as well as the Oxr1- or Ncoa7-TLDc domains (Fig. 4 *E* and *F*). In contrast, an Oxr1 mutant lacking the TLDc domain (Oxr1 ∆TLDc) did not inhibit ATPase activity (Fig. 4*F*). These data suggested that Oxr1 and Ncoa7 specifically associate with the ATP-bound A subunit in the V-ATPase V_1_ sector to inhibit ATP hydrolysis through their TLDc domains.

### Oxr1 and Ncoa7 Control the Luminal pH in the Golgi/TGN.

**Fig. 5.**
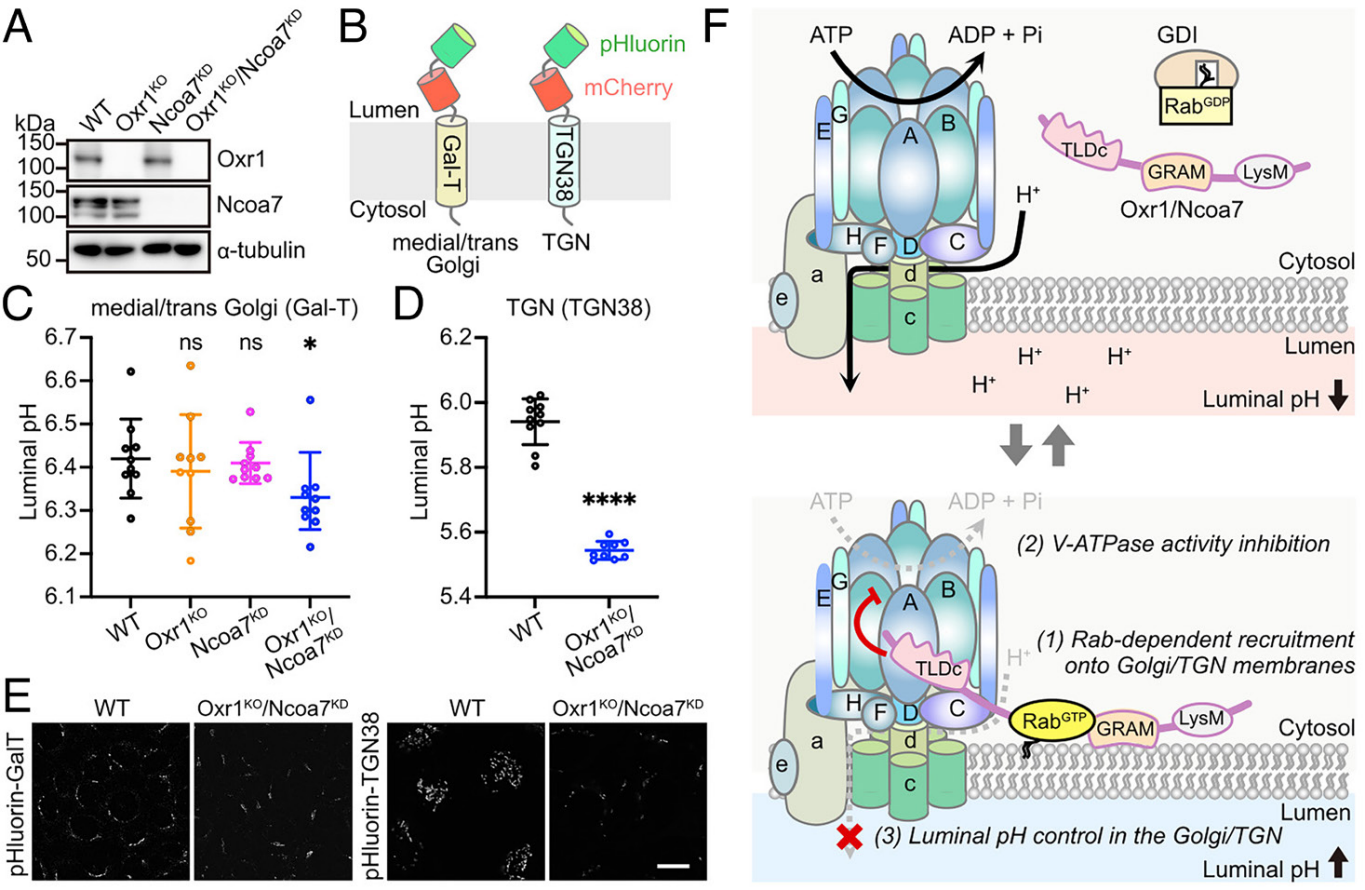
Oxr1 and Ncoa7 control luminal pH of Golgi/TGN. (*A*) Immunoblotting showing the depletion efficiencies of Oxr1 and Ncoa7 proteins in cell lysates of Oxr1^KO^, Ncoa7^KD^, and Oxr1^KO^/Ncoa7^KD^ EpH4 cells using anti-Oxr1, Ncoa7, and a-tubulin antibodies. WT cells were used as a control. (*B*) Illustration showing the fusion proteins used for luminal pH measurement in the Golgi/TGN. (*C* and *D*) Luminal pH of the medial/trans-Golgi in EpH4 cells expressing pHluorin-mCherry-GalT (*C*) and the TGN in EpH4 cells expressing pHluorin-mCherry-TGN38 (*D*). The pHluorin intensity from 10 confocal images (each containing ≥ 30 cells) was quantified and used to calculate the pH. Data expressed as means ± SD. Statistical significance analyzed using Student’s *t* test (*P < 0.05; ****P < 0.0001; ns, not significant). (*E*) Representative images of pHluorin fluorescence in WT and Oxr1^KO^/Ncoa7^KD^ EpH4 cells expressing pHluorin-mCherry-GalT or pHluorin-mCherry-TGN38 (*E*). (Scale bar, 20 µm.) (*F*) Illustrated model for the mechanism of luminal pH control in the Golgi/TGN via Oxr1 and Ncoa7.

Given their localization at the Golgi/TGN (Fig. 2) and inhibition of V-ATPase activity (Fig. 4), we hypothesized that Oxr1 and Ncoa7 may regulate luminal pH in the Golgi and TGN. To test this hypothesis, we attempted to establish Oxr1/Ncoa7-deficient EpH4 cells using the CRISPR/Cas9 system, but were only able to obtain a knockout (KO) of *Oxr1*. Thus, we employed lentivirus-mediated knockdown (KD) of *Ncoa7* transcripts in both parental WT and Oxr1^KO^ cells using a specific short hairpin (sh)RNA (Fig. 5*A*). To monitor pH within the medial/trans-Golgi and TGN, we established WT and Oxr1^KO^/Ncoa7^KD^ cells with stable expression of pHluorin- and mCherry-fused galactosyltransferase (pHluorin-mCherry-GalT) and TGN38 (pHluorin-mCherry-TGN38) proteins, respectively (Fig. 5*B*) (18, 19). The luminal pH in the medial/trans-Golgi was slightly lower in Oxr1^KO^/Ncoa7^KD^ cells than in WT, Oxr1^KO^, or Ncoa7^KD^ cells (WT, 6.41 ± 0.21; Oxr1^KO^/Ncoa7^KD^, 6.33 ± 0.14; Fig. 5 *C* and *E*). Notably, the pH in the TGN was markedly lower in Oxr1^KO^/Ncoa7^KD^ cells (5.54 ± 0.03) than in WT (5.94 ± 0.07) cells (Fig. 5 *D* and *E*). Taken together, these data suggested that Oxr1 and Ncoa7 are recruited to the Golgi/TGN membrane by GTP-bound Rab proteins, where they control the luminal pH through V-ATPase (Fig. 5*F*).

### Glycosylation Is Altered in Oxr1/Ncoa7-Deficient Cells.

**Fig. 6.**
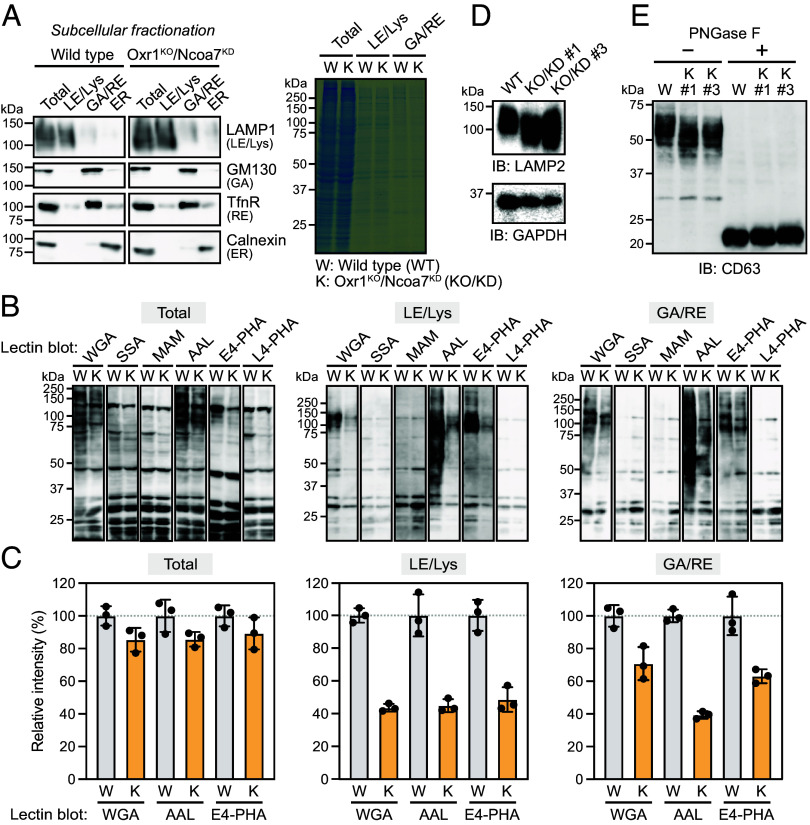
Glycosylation defects in Oxr1^KO^/Ncoa7^KD^ cells. (*A*) Immunoblotting of cell homogenates from WT and Oxr1^KO^/Ncoa7^KD^ EpH4 cells separated into LE/Lys-, GA/RE-, and ER/Mito-enriched fractions, using anti-LAMP1, GM130, TfnR, calnexin, or COXIV antibodies (*Left*). Coomassie brilliant blue-stained gel showing equivalent amounts of protein in each fraction from WT and Oxr1^KO^/Ncoa7^KD^ (K) cells (*Right*). (*B*) Lectin blotting analysis of total cell lysate (total) and LE/Lys- and GA/RE-enriched fractions from WT and Oxr1^KO^/Ncoa7^KD^ (K) cells using the following lectins: WGA, SSA, MAM, AAL, E4-PHA, and L4-PHA. (*C*) Quantification of the relative intensities of the indicated lanes from the lectin blot shown in (*B*). Data expressed as means ± SD of three independent experiments. (*D*) Immunoblotting analysis of lysates from WT and Oxr1^KO^/Ncoa7^KD^ (KO/KD) clones using anti-LAMP2 antibody. (*E*) Immunoblotting analysis of lysates from WT and Oxr1^KO^/Ncoa7^KD^ (K) cells, treated with or without PNGase F, using anti-CD63 antibody.

Glycosylation is influenced by the dysregulation of pH homeostasis in the Golgi/TGN (20), leading us to test for altered glycosylation in Oxr1^KO^/Ncoa7^KD^ EpH4 cells. The postnuclear supernatant (total) from WT or Oxr1^KO^/Ncoa7^KD^ cells was separated into LE/Lys, Golgi/recycling endosome (GA/RE), and ER/mitochondria (ER/Mito) fractions (Fig. 6*A* and *SI Appendix*, Fig. S6), which were subjected to lectin blotting. Blotting with wheat germ agglutinin (WGA; recognizes N-acetylglucosamine [GlcNAc] and sialic acid) (21), *Aleuria aurantia* lectin (AAL; fucose) (22), and *Phaseolus vulgaris* lectin (E4-PHA; bisecting GlcNAc) (23) revealed decreased glycosylation in Oxr1^KO^/Ncoa7^KD^ cells compared with that in WT cells (Fig. 6 *B* and *C*). Conversely, no differences were detected in blotting with *Sambucus sieboldiana* agglutinin (SSA; sialic acid) (24), *Maackia amurensis* lectin (MAM; sialic acid) (25), or *P. vulgaris* lectin (L4-PHA; GalNAc) (26) (Fig. 6*B*). Glycosylation defects were confirmed by immunoblotting with the highly glycosylated lysosomal membrane proteins LAMP2 and CD63, which migrated faster in sodium dodecyl sulfate (SDS)-polyacrylamide gel electrophoresis (PAGE) of Oxr1^KO^/Ncoa7^KD^ cell lysates than WT cell lysates (Fig. 6 *D* and *E*). When treatment with peptide N-glycosidase F (PNGase F) was used to remove oligosaccharides from glycoproteins, the SDS-PAGE mobility and amount of CD63 protein were similar between WT and Oxr1^KO^/Ncoa7^KD^ cells, indicating that the lower molecular weight of CD63 in Oxr1^KO^/Ncoa7^KD^ cells resulted from glycosylation defects (Fig. 6*E*). These data suggested that altered pH in the Golgi/TGN, caused by the depletion of Oxr1 and Ncoa7, affects the activity of certain glycosyltransferases, leading to partial glycosylation defects.

### Depletion of Oxr1 and Ncoa7 Causes Lysosome Dysfunction.

**Fig. 7.**
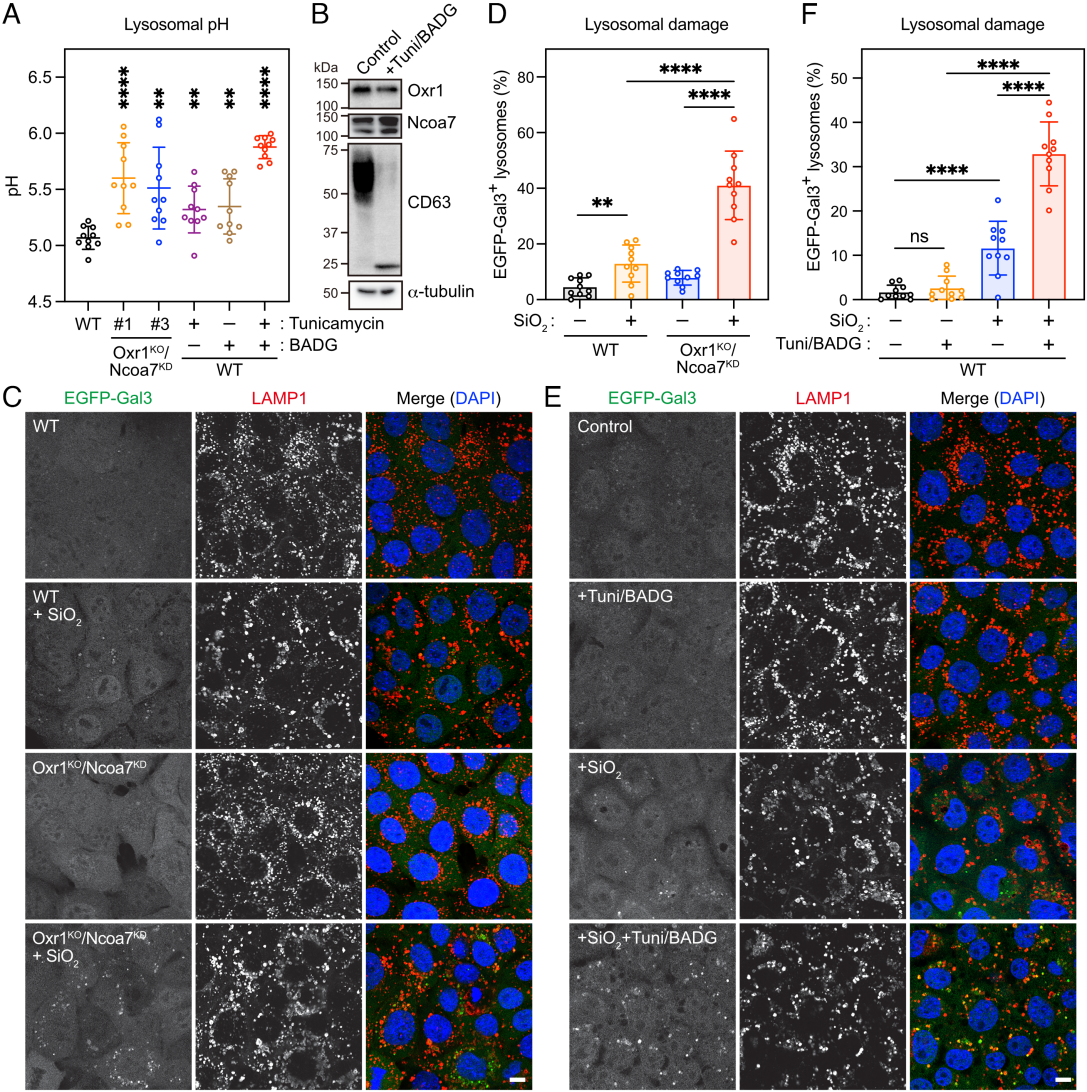
Depletion of Oxr1/Ncoa7 and inhibition of glycosylation cause lysosomal dysfunction. (*A*) Lysosomal pH of WT control, Oxr1^KO^/Ncoa7^KD^ cells, and WT cells treated with tunicamycin and/or BADG and fed with FITC-dextran. The FITC intensity from 10 images containing ≥ 30 cells was measured and used to calculate pH. Data expressed as means ± SD (n = 10 image fields). Statistical significance was analyzed using Student’s *t* test (**P < 0.005; ****P < 0.0001). (*B*) Immunoblotting of lysates from control and tunicamycin/BADG-treated cells using anti-Oxr1, Ncoa7, CD63, and a-tubulin antibodies. (*C*) Representative images of WT and Oxr1^KO^/Ncoa7^KD^ cells expressing EGFP-Gal3 and treated with or without 500 µg/mL of SiO_2_ for 24 h, followed by staining with anti-LAMP1 antibody to indicate lysosomes. (*D*) Percentage of EGFP-Gal3-positive lysosomes in (*C*). Data expressed as means ± SD. Statistical significance analyzed using Student’s *t* test (**P < 0.01; ****P < 0.0001). (*E*) Representative images of EGFP-Gal3-expressing cells treated with methanol (control) or tunicamycin/BADG, with or without further treatment with SiO_2_ for 24 h, followed by staining with anti-LAMP1 antibody. (*F*) Percentage of EGFP-Gal3-positive lysosomes in (*E*). Data expressed as means ± SD. Statistical significance analyzed using one-way ANOVA (****P < 0.0001; ns, not significant). (Scale bar, 10 µm.)

Previous studies have demonstrated an increase in lysosomal pH in neurons with *Ncoa7* KO (15). To determine whether Oxr1^KO^/Ncoa7^KD^ EpH4 cells also exhibit increased lysosomal pH, we administered fluorescein isothiocyanate (FITC)-dextran and measured fluorescence intensity in the lysosomes. Consistent with previous studies, Oxr1^KO^/Ncoa7^KD^ cells exhibited a higher lysosomal pH compared with WT cells (Fig. 7*A*). However, our findings showing that Oxr1 and Ncoa7 primarily localize at the Golgi and TGN (Fig. 2), inhibit the activity of V-ATPase in vitro (Fig. 4), and regulate luminal pH in the Golgi and TGN (Fig. 5), make it unlikely that Oxr1 and Ncoa7 directly regulate V-ATPase on the lysosomal membrane, as previously suggested (15, 27). Instead, we hypothesized that the increased lysosomal pH in Oxr1^KO^/Ncoa7^KD^ cells resulted from glycosylation defects. To test this hypothesis, we treated the cells with tunicamycin and benzyl 2-acetamido-2-deoxy-α-D-galactopyranoside (BADG) to inhibit N- and O-glycosylation. The glycosylation defect in the presence of these inhibitors was confirmed by immunoblotting with an anti-CD63 antibody (Fig. 7*B*), and the increase in lysosomal pH in tunicamycin- and/or BADG-treated cells compared with that in untreated control cells (Fig. 7*A*).

Next, we asked whether lysosomal integrity was altered in Oxr1/Ncoa7-depleted and tunicamycin/BADG-treated cells. To address this, we established parental (control) and Oxr1^KO^/Ncoa7^KD^ cells with stable expression of EGFP-Galectin-3 (Gal3). A cytosolic protein, Gal3 is recruited to permeabilized lysosomal membranes, where it coordinates with cellular components (e.g., ESCRT-III) for lysosomal repair (28). Therefore, Gal3 is commonly used as a marker for lysosomal damage (29, 30). To induce permeabilization of the lysosomal membrane, cells expressing EGFP-Gal3 were incubated with silicon dioxide (SiO_2_) nanoparticles for 24 h. After 24 h, we measured EGFP fluorescence in the lysosomes (30, 31). In the presence of SiO_2_, the increase in the number of EGFP-Gal3-positive lysosomes was markedly higher in Oxr1^KO^/Ncoa7^KD^ cells than in WT control cells (Fig. 7 *C* and *D*). We also observed that the lysosomes in cells treated with tunicamycin and BADG were more sensitive to SiO_2_ than those in untreated control cells (Fig. 7 *E* and *F*). Taken together, these data indicated that the lysosomal dysfunction in Oxr1^KO^/Ncoa7^KD^ cells, as exhibited by alterations in lysosomal pH and integrity, is caused by glycosylation defects.

## Discussion

Previous studies have revealed that Rab GTPases and their binding proteins regulate various intracellular processes on organelle membranes, including membrane trafficking (e.g. vesicle/tubule generation, motility, tethering, and fusion), lipid transfer, and remodeling of the membrane cytoskeleton (32–34). In addition to these roles in cytosolic events, we have identified a new function for Oxr1 and Ncoa7, which were identified as Rab-binding proteins that regulate proton pump activity and affect the luminal environment of organelles from the cytosolic side.

Many peripheral Rab-binding proteins are loosely attached to the membrane, cycling between the cytosol and membranous organelles in a Rab GTPase-dependent manner (35). Similarly, Oxr1 and Ncoa7 were found to bind GTP-loaded, but not GDP-loaded Rab proteins (Fig. 1), and their membrane binding was GTP-loaded Rab dependent (Fig. 3). Immunofluorescence and subcellular fractionation experiments demonstrated that Oxr1/Ncoa7 accumulated in the perinuclear Golgi/TGN area, with partial overlap with endosomes/vesicles (Fig. 2 and *SI Appendix*, Figs. S2, S3, and S6), and were present in the cytosolic fraction (*SI Appendix*, Fig. S6), suggesting that Oxr1/Ncoa7 cycle between the Golgi/TGN and cytosol. Notably, Oxr1 and Ncoa7 were not detected in the lysosomes, indicating that lysosomes are not the primary site of their functions. However, the Oxr1- and Ncoa7-binding Rab proteins identified in this study (Rab1, Rab6, Rab8, Rab10, Rab19b, Rab39a, and Rab43) reportedly localize at the Golgi, TGN, endosomes, and exocytic vesicles (2), implying that Oxr1 and Ncoa7 might also localize broadly at the Golgi and post-Golgi compartments. Thus, we presume that additional non-Rab factors may contribute to their organelle membrane-binding specificity. PIPs, which reportedly bind to several GRAM domain-containing proteins (14), are one such candidate. Indeed, a recent study showed that Ncoa7 is capable of binding PIPs, but did not fully characterize its binding specificity for different PIPs (15).

The mechanism by which the TLDc domain affects V-ATPase activity and stability remains debatable, although several studies have suggested that TLDc domain-containing proteins regulate V-ATPase activity. Based on cryogenic electron microscopy data and in vitro studies with yeast Oxr1 and V-ATPase, Khan et al. suggested two possible mechanisms: 1) yeast Oxr1 catalytically inhibits ATPase activity; and 2) Oxr1 disassembles the V-ATPase holoenzyme in a catalytically independent manner (6). Furthermore, the most recent study on yeast Oxr1 and Rtc5, another TLDc protein, showed that the vacuole fraction in *oxr1∆* and *rtc5∆* cells contained more V-ATPase than that in control cells, suggesting that these proteins disassemble V-ATPase (7). However, our in vitro experiments (Fig. 4) support the first mechanism. Our immunoprecipitation and pull-down experiments using mouse tissue lysates identified only the A subunit as an Oxr1/Ncoa7 interactor. Furthermore, our in vitro assays showed that the TLDc domains of Oxr1 and Ncoa7 directly inhibited the ATPase activity of the minimum A_3_B_3_ complex (Fig. 4), although Khan et al. suggested that the TLDc domain of yeast Oxr1 inhibits the ATP hydrolysis activity of the V_1_ sector by associating with several V_1_ subunits, particularly C, which enhances this inhibition (6). In contrast, we did not find evidence for the second mechanism involving mammalian Oxr1- and Ncoa7-mediated disassembly of V-ATPase. Our membrane fractionation and immunofluorescence experiments showed that depletion of Oxr1 and Ncoa7 did not significantly alter the membrane-bound state of the V_1_ subunits (*SI Appendix*, Fig. S7). Additionally, overexpression of Oxr1 did not affect the location of the endogenous A subunit (*SI Appendix*, Fig. S4*B*). Overall, our results suggest that Oxr1 and Ncoa7 do not mediate the disassembly of V-ATPase in mammals; if they do, such activity is undetectably lower than that in yeast cells. These discrepancies may reflect differences in the TLDc domains of TLDc family proteins. For example, a comparison study between yeast Oxr1 and mEAK7/TLDC1, another TLDc protein in mammals, showed that they bind V_1_ at different positions, suggesting that they have different affinities for V_1_ subunits (16). Furthermore, unlike Oxr1, mEAK7/TLDC1 does not affect the ATPase activity of V-ATPase in vitro (16, 36). Therefore, we speculate that mammalian Oxr1 and Ncoa7 regulate V-ATPase differently from yeast proteins and other mammalian TLDc family members.

V-ATPase is widely distributed across the Golgi to post-Golgi compartments but is particularly highly abundant in lysosomes. Consequently, the varying luminal pH values of different organelles can be explained by the abundance of V-ATPase. Our data suggest that Oxr1 and Ncoa7 are additional factors influencing the luminal pH of different organelles, with their specific localization at the Golgi/TGN playing a crucial role in maintaining the unique pH within each organelle compartment. Therefore, it will be important to assess the subcellular localization of other endogenous TLDc domain-containing proteins (e.g., TBC1D24, mEAK7/TLDC1, and c20orf118/TLDC2) to determine whether they participate in the fundamental mechanism underlying pH variation across different organelles.

Alteration of pH within the Golgi/TGN causes glycosylation defects (37), consistent with our findings of glycosylation defects in Oxr1^KO^/Ncoa7^KD^ cells (Fig. 6). Notably, proteins in the LE/Lys fraction isolated from Oxr1^KO^/Ncoa7^KD^ cells showed the strongest effects on glycosylation. The heavy glycosylation of lysosomal membrane proteins is believed to protect lysosomal membranes from hydrolytic attack by their own enzymes (38). Our data showed that lysosomal membranes in Oxr1^KO^/Ncoa7^KD^ and tunicamycin/BADG-treated cells were more sensitive to damage than those in control cells (Fig. 7), suggesting that glycosylation defects lead to lysosomal membrane fragility. Furthermore, the lysosomal pH was higher in Oxr1^KO^/Ncoa7^KD^ and tunicamycin/BADG-treated cells than in control cells (Fig. 7). This is consistent with a study showing increased lysosomal pH values in fibroblasts derived from *OXR1*-deficient patients and cortical neurons from Ncoa7-deficient mice (15, 27). However, given the inhibitory roles of Oxr1 and Ncoa7 on V-ATPase (Fig. 4) and their endogenous localization at the Golgi/TGN, but not at lysosomes (Fig. 2 and *SI Appendix*, Fig. S4), the increase in lysosomal pH is unlikely to be due to direct actions of Oxr1 and Ncoa7 on V-ATPase at the lysosomal membrane. Presumably, the increase in pH is due to leakage of protons and other ions caused by glycosylation defects, resulting in lysosomal membrane permeability. This permeability appears to be mild in Oxr1/Ncoa7-deficient cells, as indicated by the observation that EGFP-Gal3-positive lysosome numbers only increased in the presence of SiO_2_ (Fig. 7 *D*–*F*). Alternatively, based on evidence suggesting that N-glycosylation of V_0_ subunits affects V-ATPase folding, localization, and stability (39), it is possible that the incomplete glycosylation of V_0_ subunits in Oxr1/Ncoa7-deficient cells also influences the activity of V-ATPase at lysosomes.

Congenital disorders of glycosylation (CDGs), caused by mutations in genes involved in glycoprotein and lipid synthesis, produce a variety of symptoms (40). Among the many genes responsible for CDGs, several V-ATPase subunits and regulators have been reported (41–46). Previous studies on human patients with *OXR1* deficiency and *Oxr1* and *Ncoa7* KO mice have reported neurological defects and lysosomal dysfunction, which are also often found in CDG patients (40, 47, 48). Such findings, along with our data, highlight the implications of OXR1 and NCOA7 in patients with CDGs.

In conclusion, Oxr1 and Ncoa7 regulate V-ATPase activity and modulate pH within the Golgi apparatus and TGN, creating an optimal environment for enzymes involved in glycosylation. Our findings also demonstrate that increases in luminal pH and fragility of lysosomes result from organelle dysfunction caused by the depletion of Oxr1 and Ncoa7, and the inhibition of glycosylation. Further studies exploring the impacts of these proteins on other organelles and cellular events are required to fully elucidate the pathology of Oxr1 and Ncoa7 deficiencies.

## Materials and Methods

### Antibodies, Lectins, and Reagents.

For immunofluorescence and immunoblotting assays, rabbit anti-Oxr1 (Proteintech, 13514-1-AP), Ncoa7 (Atlas Antibodies, HPA030292 or Proteintech, 23092-1-AP), CD63 (abcam, EPR21151, ab217345), ATP6V1A (abcam, EPR19270, ab199326), and rat anti-LAMP1 and LAMP2 (Developmental Studies Hybridoma Bank, H4A3) antibodies were used. For immunofluorescence, mouse anti-Syntaxin-6 (BD Bioscience, AB_397966), sheep anti-GRASP55 (49), and goat anti-Sorting nexin-1 (Santa Cruz Biotechnology, T-19, sc-10609) antibodies were used. For immunoblotting, rabbit anti-ATP6V1B2 (Proteintech, 15097-1-AP), ATP6V1C1 (Proteintech, 16054-1-AP), ATP6V1E1 (Proteintech, 15280-1-AP), ATP6V1G2 (Proteintech, 25316-1-AP), ATP6V0A1(a1, Proteintech, 13828-1-AP), GFP (50), calnexin-CT (Stress Marq, SPC-108), Rab1a (Santa Cruz Biotechnology, C-19, sc-311), Rab6 (Sigma-Aldrich, H7171-3G3), Rab10 (Abcam, MJF-R23), mouse anti-alpha tubulin (Sigma, DM1A, T6199), FLAG (M2, Sigma, F1804), and Rab8 (BD Biosciences, 610844) antibodies. The biotinylated lectins WGA (J1001016), SSA (J1001014), MAM (J1001009), AAL (J1001001), E4-PHA (J1001010), and L4-PHA (J1001011) were purchased from J-CHEMICAL. Donkey anti-rabbit and anti-mouse antibodies conjugated to Alexa-488, and donkey anti-rabbit, anti-mouse, and anti-goat antibodies conjugated to Alexa-568 were purchased from Thermo Fisher Scientific. Horseradish peroxidase (HRP)-conjugated donkey anti-rabbit and anti-mouse antibodies, and HRP-streptavidin were purchased from Jackson ImmunoResearch.

All reagents were purchased from FUJIFILM-Wako chemicals, except for saponin (Nacalai Tesuque, 30502-42), nigericin sodium salt (Sigma-Aldrich, N7413), monensin sodium salt (Sigma-Aldrich, M5273), BADG (Sigma-Aldrich, 200100), tunicamycin (Selleck Chemicals, S7894), FITC-dextran (Tokyo Industrial Chemistry, F0918), SiO_2_ (Sigma-Aldrich, 637238), and [γ-^32^P]ATP (Revvity Health Sciences, BLU002Z).

### Plasmids.

All Rab constructs for expression in mammalian, bacterial, and yeast two-hybrid systems were prepared as previously described (51, 52). The cDNAs encoding *Mus musculus* Oxr1 (NM_001358977.1), Ncoa7 (NM_172495.6), ATP6V1A (BC038392.1), and ATP6V1B2 (BC012497) were amplified by KOD Plus-Ver.2 (TOYOBO) using 17-d embryo Marathon-Ready mouse cDNA (TaKaRa Clontech). *Homo sapiens* Rab GDI-alpha cDNA (D45021.1) was amplified from human fetal Marathon-Ready cDNA (TaKaRa Clontech) using KOD One DNA polymerase (TOYOBO). The full-length and deletion mutants of the Oxr1 and Ncoa7 cDNAs were subcloned into plasmids pACT2 (yeast two-hybrid), or pFAT2 or pQE32 (bacteria). Oxr1, ATP6V1A, and ATP6V1B2 were subcloned into pcDNA5 FRT/TO-FLAG or -EGFP plasmids. RabGDI-alpha and GFP were subcloned into pFAT2. The EGFP-Gal3 fragment was amplified by PCR using pEGFP-hGal3 [a gift from Dr. Tamotsu Yoshimori; Addgene plasmid # 73080 (30)] as template, and then subcloned into pMSCV-hygro. To generate pHluorin-GalT, EGFP was replaced with pHluorin at the *Bam*HI and *Not*I sites [a gift from Dr. Jennifer Lippincott-Schwartz; Addgene plasmid #11929 (53)]. The pHluorin-TGN38 plasmid was generated by subcloning the fusion construct of the signal sequences of interleukin-2 (IL-2), pHluorin, and the region corresponding to the C-terminal 56 amino acids of rat TGN38 in pcDNA3.1. *T. thermophilus* subunits A and B were amplified by PCR from pG3-FABD [a gift from Dr. Ken Yokoyama (54)], and then subcloned into pETDuet-1. The Oxr1 CRISPR plasmid was constructed by subcloning a DNA oligonucleotide encoding a single-guide RNA (5′-tggactccactgaatgaaga-3′) subcloned at the *Bbs*I site of pX330. The lentiviral plasmid pLKO.1, which encodes shRNA against *Ncoa7* (TRCN0000190323 and TRCN0000201060), was purchased from Sigma-Aldrich.

### Protein Expression and Purification.

To produce His_6_ and His_6_-GST-fusion proteins, the corresponding expression plasmids were transfected into Rosetta 2(DE3)pLys cells (Novagen). The bacteria were then incubated at 18 °C for 16 h in the presence of 0.25 mM isopropyl β-D-thiogalactopyranoside (IPTG). Cell lysates were incubated with Ni-NTA agarose (Qiagen) for 2 h and bound proteins were eluted with 200 mM imidazole. Proteins were dialyzed against phosphate-buffered saline (PBS), snap-frozen in liquid N_2_, and stored at −80 °C until use.

### Pull-Down and Immunoprecipitation.

GST extraction using mouse brain lysates and the His_6_-GST-Rab6 protein was carried out as previously described (55). For GST pull-down using His_6_-GST-Oxr1 or -Ncoa7 fusion proteins, mouse brain was lysed with RIPA buffer (10 mM Tris-HCl, pH 7.5, 150 mM NaCl, 1% Triton X‐100, 0.1% Na‐SDS, and 1% sodium deoxycholate containing protease inhibitors) and diluted 1:50 in NL100 (20 mM HEPES-NaOH, pH 7.5, 100 mM NaCl, 5 mM MgCl_2_, and 0.1% Triton X‐100). The lysate containing 1 mg of protein was incubated with 10 µg of GST-fused protein bound to glutathione-Sepharose beads (Cytiva) for 1 h at 4 °C. The beads were then washed with NL100 three times, and the proteins were analyzed by immunoblotting using antibodies against V-ATPase subunits. For pull-down using purified Oxr1 and Rab6 proteins, 1 µg of His_6_-tagged Oxr1 was incubated with 5 µg of immobilized GDP-loaded T27N, GTP-loaded Q72A mutant, or GTP-loaded WT GST-fused Rab6 in NL 100 buffer for 1 h at 4 °C. The proteins were eluted with SDS-PAGE sample buffer and analyzed by immunoblotting using anti-Oxr1 antibody. For pull-down using a FLAG-Rab-expressing cell lysate and His_6_-GST-Oxr1 and -Ncoa7 fusion proteins, 1 × 10^6^ HEK293 cells expressing a EGFP-Rab GTP-restricted mutant were lysed with 500 µL of NL100 and incubated with GST-fusion proteins bound to glutathione beads. For pull-down assays using purified Oxr1, Ncoa7, and ATP6V1A, HEK293 cells expressing FLAG-tagged ATP6V1A were lysed with NL100 and then incubated with FLAG M2 agarose beads for 2 h at 4 °C. The beads were washed with NE500 (20 mM HEPES-NaOH, pH 7.5, 500 mM NaCl, 20 mM EDTA, and 0.1% Triton X‐100) to remove the adenine nucleotides from ATP6V1A. The nucleotide-free ATP6V1A protein was immobilized with agarose beads and then incubated with His_6_-GST-Oxr1 and -Ncoa7 fusion proteins in NL100 containing 100 µM of ADP or AMP-PNP for 1 h at 4 °C. Bound proteins were analyzed by immunoblotting using anti-GST and anti-FLAG M2 antibodies. For immunoprecipitation of Oxr1, 10 mg of mouse brain lysate dissolved in NL100 was incubated with 5 µg of affinity-purified anti-Oxr1 antibody or control IgG for 2 h at 4 °C, followed by incubation with protein G Sepharose (Cytiva) for 1 h. The sepharose beads were then washed with NL100 three times, subjected to SDS-PAGE, and further analyzed by mass spectrometry.

### Mass Spectrometry.

The pull-down or immunoprecipitated protein samples were separated on NuPAGE Novex Bis-Tris gels (Thermo Fisher Scientific) and then silver stained as previously described (50). The bands were excised from the gel and the proteins were reduced, alkylated, and digested with trypsin in Tris-buffered saline for 16 h at 37 °C. Proteins were analyzed using a Q-Exactive mass spectrometer (Thermo Fisher Scientific) at the Osaka University Center for Medical Research and Education. Searches were conducted using the Mascot server (v2.3; Matrix Science) and the International Protein Index (mouse, v3.77 or v3.87; EMBL-EBI) databases.

### Yeast Two-Hybrid Assay.

Yeast two-hybrid assays were performed as previously described (56). The PJ96-4A strain was cotransformed with Gal4-AD (pACT2-Oxr1 or its deletion construct) and Gal4-BD (pFBT9-Rab) expression plasmids. The transformants were grown on synthetic complete medium lacking Leu/Trp (SC-LW) for 3 d at 30 °C. Five independent colonies were restreaked onto SC–LW and quadruple dropout medium lacking Leu/Trp/His/Ade (QDO), and then incubated for 3 d at 30 °C.

### Subcellular Fractionation.

A total 4 × 10^7^ EpH4 control or Oxr1^KO^/Ncoa7^KD^ cells plated the day before were washed once with 20 mL PBS. Cells were then scraped into PBS, pelleted by centrifugation at 1,000×*g* for 5 min, and resuspended in 500 µL homogenization buffer (25 mM Tris-HCl, pH 7.3, 130 mM KCl, and 5 mM MgCl_2_) containing protease inhibitors. This mixture was homogenized by 20 passages through a 27-gauge needle, and then centrifuged twice at 1,000×*g* for 5 min to collect the postnuclear supernatant (PNS). The PNS was layered on top of a step density gradient comprising 3 mL 5%, 2 mL 10%, 2 mL 12.5%, 1 mL 15%, 1 mL 20%, 2 mL 25%, and 0.5 mL 40% Histodenz (Sigma-Aldrich) in homogenization buffer in a 13.2-mL Ultra-Clear centrifuge tube (Beckman). The gradient was then centrifuged at 100,000×*g* using a SW41Ti rotor (Beckman) for 4 h at 4 °C. Twelve fractions were collected from top to bottom. All fractions were analyzed by immunoblotting using antibodies against Oxr1, Ncoa7, GM130, TfnR, LAMP1 or LAMP2, calnexin, COXIV, GAPDH (cytosol), and ATP6V01. The 4th to 6th fractions from the top (1st) fraction were collected as being enriched in LE/Lys, the 9th fraction in Golgi/RE, and the 12th fraction in ER. The PNS-, Golgi/RE-, Lys-, and ER/Mito-enriched fractions were analyzed by lectin blotting.

### Rab-GDI Extraction Assay.

EpH4 cells grown to 100% confluency in a 10-cm dish were homogenized with 10 passages through a 27-gauge needle in buffer (5 mM MgCl_2_, 25 mM Tris-HCl (pH 7.3) containing 1 mM DTT and 250 mM sucrose. The homogenates were then centrifuged twice at 1,000×*g* for 5 min to collect the PNS. Next, 500 µL PNS was placed on 30 µL of a 2.5 M sucrose cushion and then centrifuged at 100,000×*g* for 30 min using a TLA 110 rotor (Beckman Coulter). After the supernatant was removed, the pellet containing the membrane and the sucrose cushion (30 µL) was resuspended in 250 µL of the same buffer. Next, 1 mM EDTA and 1 mM GDP were added to 200 µL of the membrane suspension and incubated at 37 °C for 45 min. in the presence of 10 µg of GST or GST-GDI protein. After incubation, the reaction mixture was centrifuged at 100,000×*g* for 30 min to separate the supernatant and membrane pellet fractions. Each fraction was analyzed by immunoblotting using various antibodies against Rab.

### Treatment with PNGase F.

EpH4 control or Oxr1^KO^/Ncoa7^KD^ cells were lysed using a buffer (10 mM Tris-HCl pH 7.8, 0.5 M NaCl, 1 mM ethylenediaminetetraacetic acid, 1% NP-40) containing protease inhibitors. After centrifugation at 15,000×*g* for 10 min at 4 °C, the supernatant was boiled in 0.1 M 2-mercaptoethanol and 0.5% SDS for 10 min. After boiling, 25 µg of protein was incubated for 16 h at 37 °C in 100 mM Tris-HCl (pH 8.6), 1% NP-40, and 40 mU/mL PNGase F (TAKARA). The samples were subjected to SDS-PAGE and analyzed by immunoblotting using an anti-CD63 antibody.

### ATPase Assay.

To measure ATP hydrolysis, 100 pmol of the A subunit or A_3_B_3_ hexamer of *T. thermophilus* V-ATPase was mixed on ice with 10 μL of 10× assay buffer (500 mM HEPES-NaOH, pH 6.8, 10 mM DTT, 2 mg/mL bovine serum albumin), 10 μL of 10 mM EDTA (pH 8.0), 5 μL of 1 mM Mg^2+^‐ATP, and 2 μL of [γ‐^32^P]ATP (10 mCi/mL; 6000 Ci/mmol), and adjusted to 100 μL with dH_2_O. The ATP‐loading reaction was performed for 15 min at 30 °C, then further incubated in the presence of 10 pmol of GST-GFP, Oxr1, or Ncoa7 proteins at 30 °C, taking 5-μL samples in duplicate at each time point (0, 15, 30, 45, 60, 75, and 120 min). The 5-μL samples were immediately added to 795 μL of 5% activated charcoal slurry (wt/vol) in 50 mM NaH_2_PO_4_ and left for 1 h on ice, then centrifuged at 16,000×*g* in a benchtop microcentrifuge to pellet the charcoal. The supernatant (400 µL) was collected, mixed with 4 mL of Ultima Gold scintillation liquid (PerkinElmer), and counted. A 2.5-μL aliquot of the assay mix was also separately counted for scintillation to calculate the specific activity of the reaction.

### Cell Culture, Transfection, and Infection.

EpH4, Neuro-2A, and HEK293FT cells were cultured in DMEM containing high glucose, L-glutamine, phenol red, sodium pyruvate, and 10% fetal bovine serum at 37 °C and 5% CO_2_. For plasmid transfection, Lipofectamine 2000 (Thermo Fisher Scientific) was used in accordance with the manufacturer's instructions. To obtain a cell line with stable expression of pHluorin-GalT and pHluorin-TGN38, transfected cells were kept in a medium containing 1.2 mg/mg of G418. The cells were then seeded at one cell per well in 96-well plates to obtain clones, which were selected using a fluorescence microscope. A lentivirus harboring shRNA against *Ncoa7* was produced as previously described (50). Retroviruses harboring EGFP-Gal3 (TaKaRa Bio) were produced in accordance with the manufacturer’s instructions. EpH4 cells were infected with these recombinant lentivirus or retrovirus vectors and cultured in medium containing 100 µg/mg hygromycin B for 10 d. Depletion of Ncoa7 or expression of the EGFP-Gal3 protein were examined by immunofluorescence and immunoblotting analyses.

To inhibit N- and O-glycosylation, cells expressing EGFP-Gal3 were incubated with medium containing 0.02 µg/mL tunicamycin and 1 mM BADG for 24 h. To measure lysosomal damage, cells were incubated in medium containing 500 µg/mL SiO_2_ for 24 h.

### Immunofluorescence.

Cells on coverslips were fixed with 3% paraformaldehyde in PBS, permeabilized with 0.05% saponin in PBS, and incubated with primary antibodies for 1 h at room temperature. The cells were then washed three times with PBS and incubated with the appropriate Alexa Fluor–conjugated secondary antibody and DAPI for 1 h. The coverslips were mounted using Mowiol. To stain Oxr1, Oxr1^KO^ cells were fixed with 3% paraformaldehyde for 15 min and sonicated three times for 30 s, with 30-s intervals. Cell debris was washed with PBS followed by incubation with anti-Oxr1 antibody for 24 h at 37 °C. After centrifugation, the supernatant containing the Oxr1 antibody was used for staining. Images were acquired using an FV1000-D confocal microscope with UPLSAPO 60× object glass (NA 1.42), UPLSAPO 40× object glass (NA 0.95), and FluoView software (all from Olympus).

### Measurement of pH.

To measure the luminal pH of Golgi and TGN, EpH4 cells expressing pHluorin-GalT and pHluorin-TGN38 were used. To measure lysosomal pH, cells were incubated with 100 µg/mL of FITC-dextran-containing medium for 24 h. For the calibration curves, cells with pHluorin fusion protein expression or FITC-dextran internalization were plated on glass-bottomed dishes and incubated in calibration buffer (140 mM KCl, 2 mM CaCl_2_, 1 mM MgSO_4_, 1.5 mM K_2_HPO_4_, 10 mM glucose, 10 μM nigericin, 10 μM monensin, 10 mM MES, and 10 mM HEPES) adjusted to various pH values (5.5, 6.0, 6.25, 6.5, 6.75) for 10 min at room temperature. Ten confocal microscopy images containing ≥ 30 cells were captured at each pH value. The fluorescence intensity of pHluorin or FITC was quantified using ImageJ. Oxr1^KO^, Ncoa7^KD^, and Oxr1^KO^/Ncoa7^KD^ cells expressing pHluorin-GalT and pHluorin-TGN38 were seeded on a separate plate and washed three times with phenol red-free DMEM and cultured for 10 min at room temperature. The fluorescent signal was captured and analyzed as described above.

## Supplementary Material

Appendix 01 (PDF)

## Data Availability

All study data are included in the article and/or *SI Appendix*.
