# Supplementary material for: Oxr1 and Ncoa7 regulate V-ATPase to achieve optimal pH for glycosylation within the Golgi apparatus and trans-Golgi network
Source: Proc Natl Acad Sci U S A. 2025 May 30;122(22):e2505975122. doi: 10.1073/pnas.2505975122 (PMC12146697; doi:10.1073/pnas.2505975122)
Supplement: Supplementary file 1 — Appendix 01 (PDF) [file pnas.2505975122.sapp.pdf]

Supporting Information for

Oxr1 and Ncoa7 regulate V-ATPase to achieve optimal pH for glycosylation within the Golgi apparatus and trans-Golgi network

Shin-ichiro Yoshimura*, Tomoaki Sobajima, Masataka Kunii, and Akihiro Harada

Department of Cell Biology, Graduate School of Medicine, Osaka University, 2-2, Yamadaoka, Suita, Osaka 565-0871, Japan

*Shin-ichiro Yoshimura

Email: shyoshimura@acb.med.osaka-u.ac.jp

This PDF file includes:

Figures S1 to S7

Tables S1 to S2

Figure S1

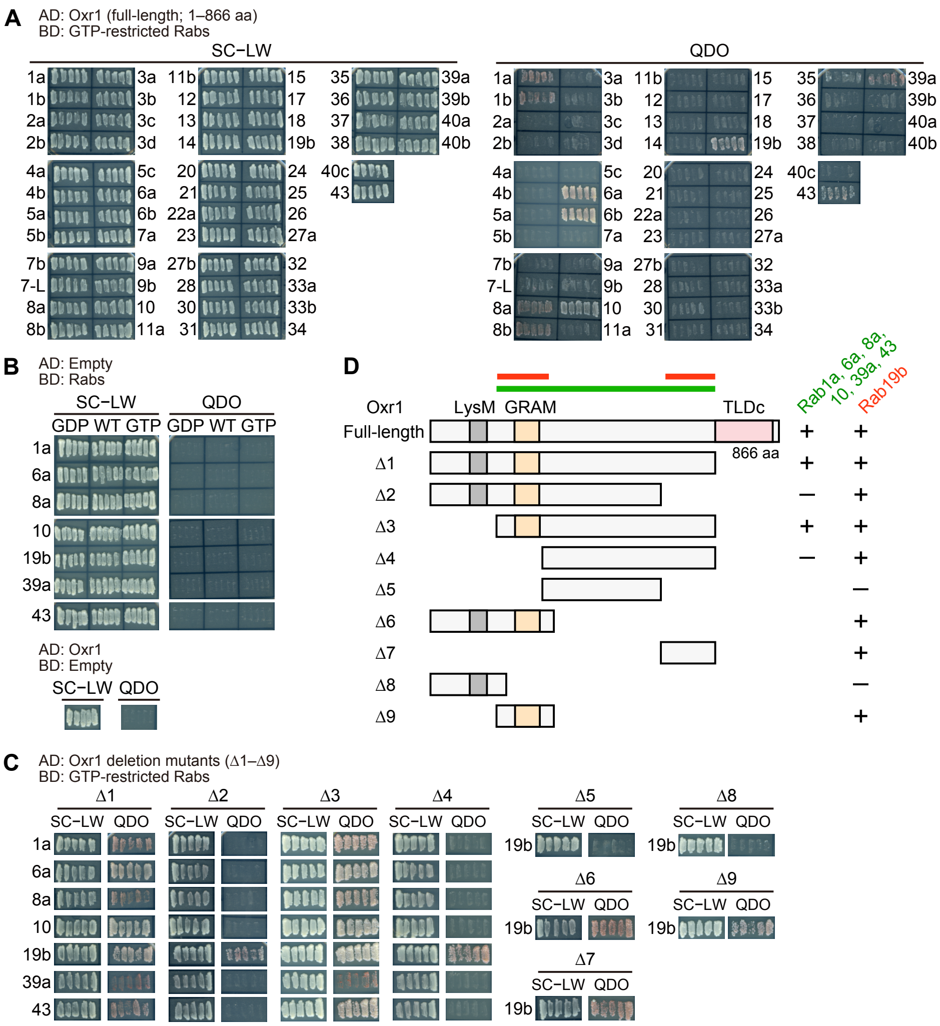

Figure S1. Yeast two-hybrid assay of Oxr1 interaction with a panel of Rab proteins.

(A) Yeast two-hybrid assay of Oxr1 binding to 58 GTP-restricted Rab mutants using yeast grown on SC-LW or QDO media. (B) Control yeast two-hybrid assay using either Oxr1 or WT, GTP- or GDP-restricted mutants of Rab, showing no growth on QDO. (C) Yeast two-hybrid assay of Oxr1 deletion mutants against Rab1a, Rab6a, Rab8a, Rab10, Rab19b, Rab39a, and Rab43. (D) Schematic drawing of full-length Oxr1 and deletion mutants used in the experiments, with summarized results for each construct from (C) shown at the right. Red lines indicate the region necessary for binding Rab19b, while the green line indicates the region necessary for binding Rab1a, Rab6a, Rab8a, Rab10, Rab39a, and Rab43.

Figure S2

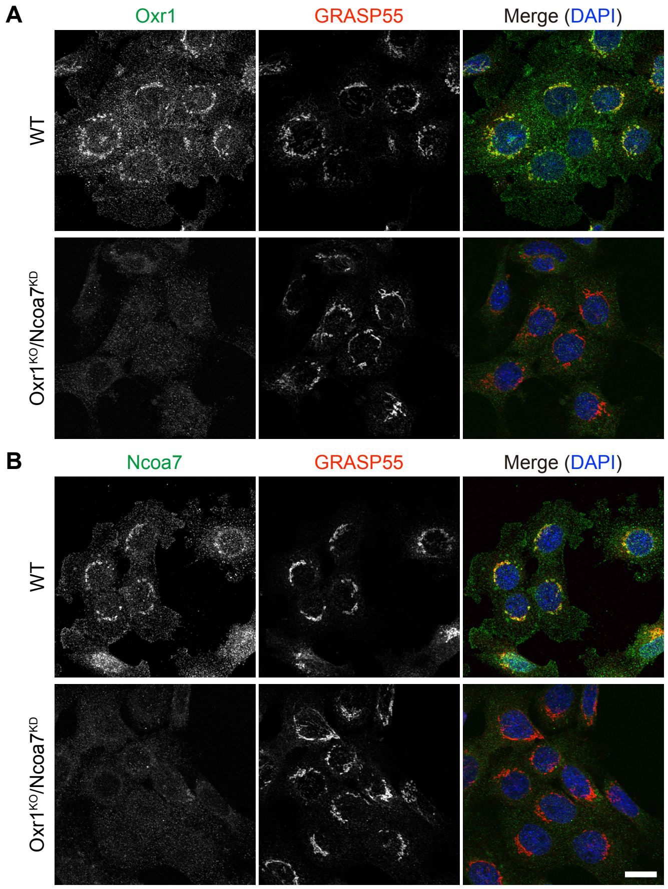

Figure S2. Assessment of the specificities of antibodies against Oxr1 and Ncoa7.

Co-staining of WT and Oxr1^{KO}/Ncoa7^{KD} EpH4 cells with anti-Oxr1 or anti-Ncoa7, along with anti-GRASP55 antibodies. Scale bar: 20 μm.

Figure S3

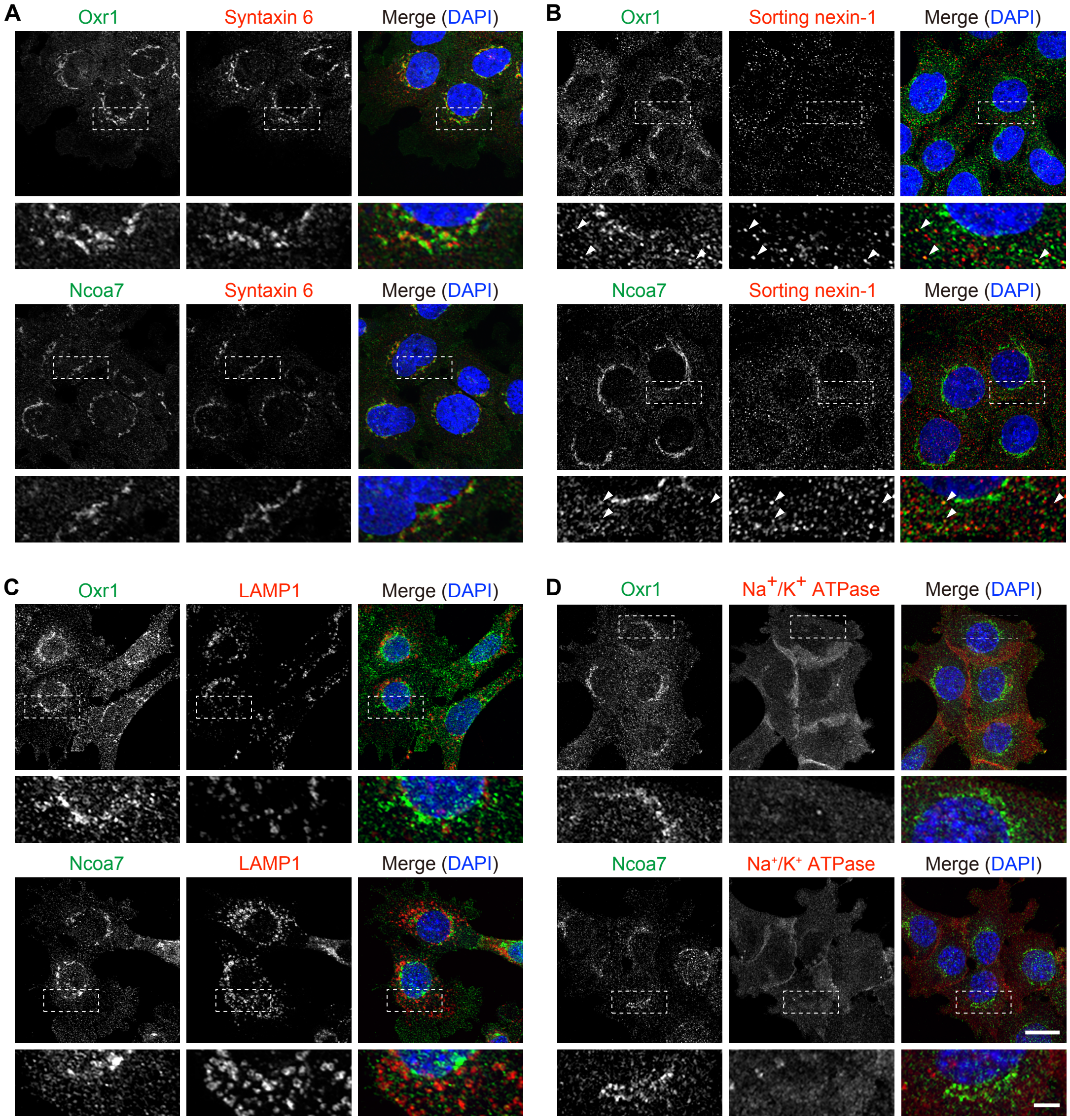

Figure S3. Subcellular localization of Oxr1 and Ncoa7.

Staining of EpH4 cells with anti-Oxr1 or Ncoa7 antibodies. The TGN (A), sorting endosomes (B), late endosomes/lysosomes (C) and plasma membrane (D) are indicated by co-staining with anti-syntaxin-6, anti-sorting nexin-1, anti-LAMP1, and Na^{+}/K^{+} ATPase antibodies, respectively. The area surrounded by the dashed line was magnified as shown under the main image. Panels below each image show magnification of the areas indicated by dashed lines. Arrowheads indicate Oxr1/Ncoa7 localization at endosomes. Scale bars: 20 µm (main image); 5 µm (magnified image).

Figure S4

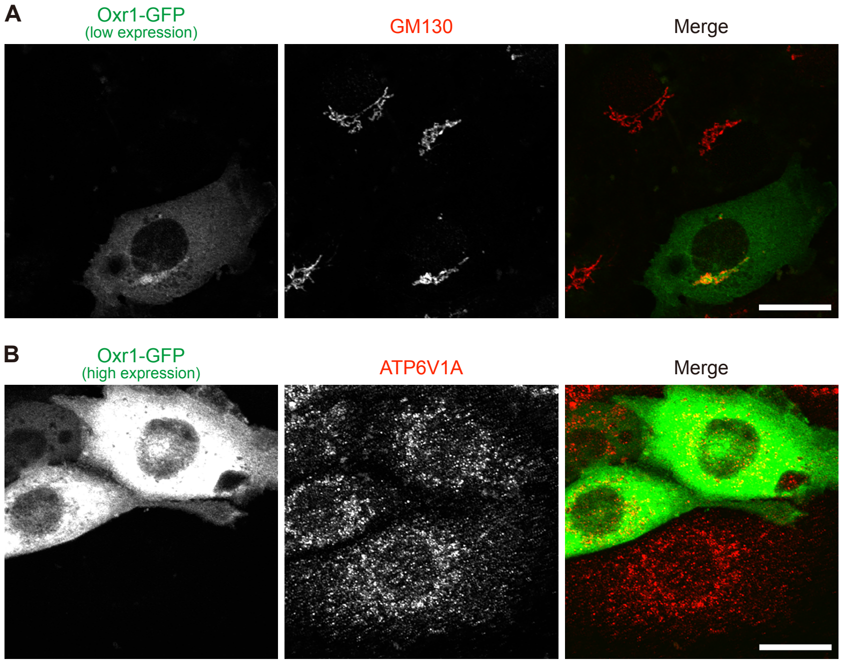

Figure S4. Localization of exogenously expressed Oxr1.

(A and B) Representative immunostaining images of EpH4 cells with low (A) or high (B) expression of Oxr1 C-terminally fused EGFP (Oxr1-GFP) using anti-GRASP55 (A) or ATP6V1A (B) antibodies. Scale bars: 20 μm.

Figure S5

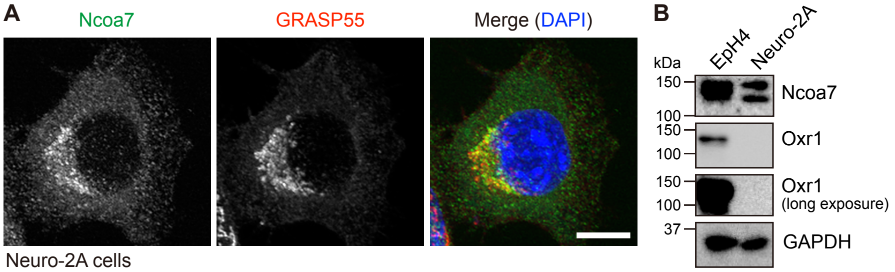

Figure S5. Expression and localization of Oxr1 and Ncoa7 in Neuro-2A cells.

(A) Co-immunostaining of Neuro-2A cells using anti-Ncoa7 and anti-GRASP55 antibodies. Nuclear staining indicated by DAPI. Scale bar: 10 µm. (B) Immunoblotting analysis of EpH4 and Neuro-2A cell lysates using anti-Oxr1 and Ncoa7 antibodies, with anti-GAPDH antibody used as a control.

Figure S6

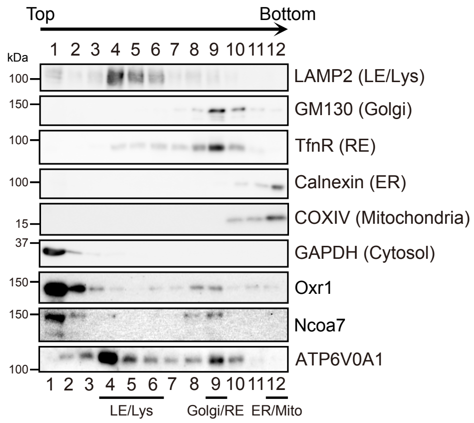

Figure S6. Subcellular fractionation by the density gradient centrifugation.

Immunoblotting analysis of 12 subcellular fractions collected from top to bottom using antibodies against the following: Oxr1, Ncoa7, GM130 (Golgi), TfnR (RE), LAMP2 (LE/Lys), calnexin (ER), COXIV (mitochondria), GAPDH (cytosol), and ATP6V01. Briefly, the 500-µl PNS derived from an EpH4 cell homogenate was layered on top of a step density gradient comprising 3 mL 5%, 2 mL 10%, 2 mL 12.5%, 1 mL 15%, 1 mL 20%, 2 mL 25% and 0.5 mL 40% Histodenz and centrifuged.

Figure S7

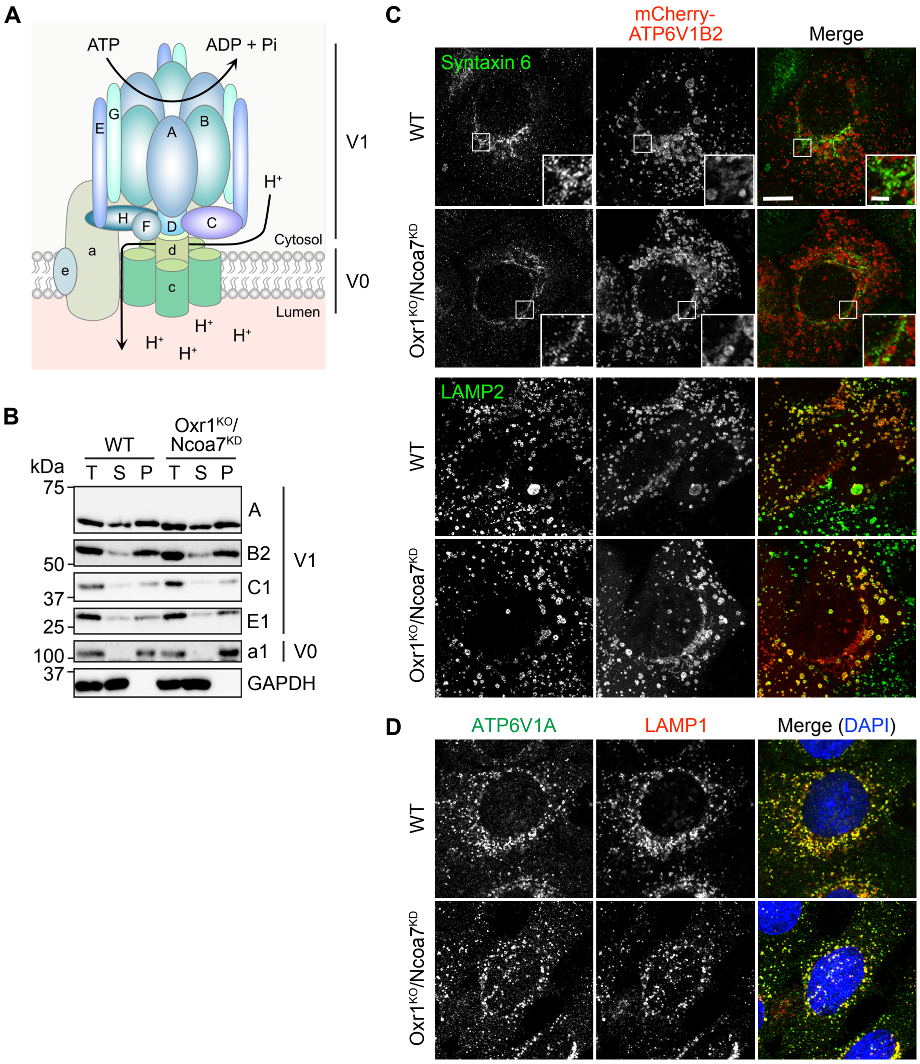

Figure S7. Subcellular distribution of V-ATPase in Oxr1^{KO}/Ncoa7^{KD} cells.

(A) Illustration of V-ATPase. (B) SDS-PAGE and immunoblotting analysis of the total post-nuclear supernatant (T), cytosolic supernatant (S), and membrane pellet fraction (P) from WT and Oxr1^{KO}/Ncoa7^{KD} EpH4 cells using antibodies against V_1 sector subunits (ATP6V1A, ATP6V1B2, ATP6V1C1, ATP6V1E1), the V_0 sector subunit ATP6V0A1, and GAPDH as a cytosolic protein control. (C) Immunostaining of WT and Oxr1^{KO}/Ncoa7^{KD} EpH4 cells showing subcellular localization of the exogenously expressed mCherry-fused ATP6V1B2 subunit. TGN and lysosomes are indicated by Syntaxin-6 and LAMP1, respectively. (D) Co-immunostaining of WT and Oxr1^{KO}/Ncoa7^{KD} EpH4 cells using anti-ATP6V1A and LAMP1 antibodies. Nuclear staining indicated by DAPI. Scale bars: 10 μm (main image); 2 μm (inset).

Table S1. **Mass spectrometric analysis of proteins identified via co-immunoprecipitation using anti-Oxr1 antibody.**

Identified Proteins (19)	Accession Number	MW (kDa)	Total Spectrum Count
Oxr1	Q4KMM3	96	126
Atp6v1a	P50516	68	74
Pacsin2	Q3TDA7	56	33
Tuba1a	P68369	50	18
Camk2b	P28652	60	14
Fus	P56959	53	13
Ddx5	Q61656	69	12
Ddb1	Q3U1J4	127	8
Klhl22	H9KV05	74	8
Hnrnpk	B2M1R6	49	7
Acap2	Q6ZQK5	87	7
Dnm1l	E9PUD2	80	6
Ubash3b	Q8BGG7	71	4
Stat5a	P42230	91	3
Tnpo2	Q99LG2	100	3
Cct8	P42932	60	3
Ogt1	Q8CGY8	117	3
Dcp1a	Q91YD3	65	3
Adrbk1	Q3U1V3	80	3

Table S2. Mass spectrometric analysis of proteins identified via pull-down assay using full-length GST-Oxr1 fusion protein.

Identified Proteins (22)	Accession Number	MW (kDa)	Total Spectrum Count
Atp6v1a	P50516	68	58
Hspa8	P63017	71	56
Stxbp1	O08599	68	26
Hspa9	P38647	73	15
Lmnb1	P14733	67	18
Pabpc1	P29341	71	14
Dpysl2	O08553	62	9
Hspa5	P20029	72	14
No66	Q9JJF3	68	9
Anxa6	F8WIT2	75	9
Ncoa5	Q91W39	65	5
Ddx3y	Q62095	73	10
Tkt	P40142	68	4
Syncrip	G3UZ48	52	8
Lmnb2	P21619	67	10
Rpn1	Q91YQ5	69	5
Hspa1l	P16627	71	10
Ckap4	Q8BMK4	64	5
Camkv	A0A0A6YW88	52	3
Ddx5	Q61656	69	4
Eif3d	O70194	64	3
Kars	Q8R2P8	71	3
